# MicroRNA-212 inhibits the proliferation, migration and invasion of renal cell carcinoma by targeting X-linked inhibitor of apoptosis protein (XIAP)

**DOI:** 10.18632/oncotarget.20786

**Published:** 2017-09-08

**Authors:** Chaohui Gu, Zhiyu Wang, Zhibo Jin, Guanru Li, Yiping Kou, Zhankui Jia, Jinjian Yang, Fengyan Tian

**Affiliations:** ^1^ Department of Urology and Henan Institute of Urology, Zhengzhou Key Laboratory for Molecular Biology of Urological Tumor Research, The First Affiliated Hospital of Zhengzhou University, Zhengzhou, Henan 450052, P.R. China; ^2^ Department of Pediatrics, The First Affiliated Hospital of Zhengzhou University, Zhengzhou, Henan 450052, P.R. China

**Keywords:** miR-212, XIAP, renal cell carcinoma, tumor growth, invasion

## Abstract

MicroRNAs have been found to be critical regulator of cancer cell biology. MicroRNA-212 (miR-212) was identified to be a critical cancer-associated microRNA playing either oncogenic functions or tumor suppressive roles in different types of human cancers. In this study, we found that the level of miR-212 in renal cell carcinoma (RCC) tissues was significantly lower than that in adjacent non-tumor tissues. Decreased level of miR-212 was associated with advanced T stage and TNM stage of RCC. The expression of miR-212 was decreased in RCC cell lines as compared with the HK-2 cell line. Overexpression of miR-212 inhibited cell viability, proliferation, migration and invasion of CAKI-2 cells. Knockdown of miR-212 increased cell viability and proliferation, migration and invasion of ACHN cells. *In vivo* experiments showed that miR-212 inhibited the proliferation and promoted the apoptosis of ACHN cells in nude mice and thus inhibited the *in vivo* tumor growth of CAKI-2 cells. Furthermore, we confirmed that X-linked inhibitor of apoptosis protein (XIAP) was the downstream target of miR-212. The expression level of miR-212 was negatively correlated with XIAP expression in RCC tissues. Moreover, XIAP mediated the tumor suppressive roles of miR-212 in RCC. Finally, we demonstrated that the aberrant expression of miR-212 and XIAP was evidently correlated with poor prognosis of RCC patients. In all, miR-212 can act as a prognostic biomarker for RCC patients and inhibits the growth and metastasis of RCC cells by inhibiting XIAP.

## INTRODUCTION

Renal cell carcinoma (RCC), accounting for 2% to 3% of adults malignant tumors, is the most common type of cancer in adult kidney [1]. Clear cell renal cell carcinoma (ccRCC), compared with other three histologic subtypes including papillary RCC, chromophobe RCC, and oncocytomas, is the most aggressive and frequent RCC histologic subtype, accounting for around 80% of RCC cases [2]. RCC in early stage is curable by surgical resection, but around 20% RCC patients have the occurrence of metastasis at the time of diagnosis [3]. For RCC patients with metastasis, the 5-year survival rate was even lower than 10%. Additionally, 30% of RCC patients with localized RCC eventually developed distant metastases after receiving curative surgical resection [4]. However, it remains largely unknown regarding the molecular mechanisms of the progression of RCC. Therefore, it is of great importance to investigate the mechanisms of RCC growth and metastasis to identify novel early-diagnosis biomarkers and therapeutic targets for RCC patients.

MicroRNAs (miRNAs), a cluster of 20–24 nucleotide noncoding RNAs, post-transcriptionally inhibit the expression of targeted gene by preventing the translation of mRNAs or promoting the degradation of mRNAs [5, 6]. Abnormal expression and function of miRNAs have been demonstrated to play important roles in the tumorigenesis and progression of many human cancers including RCC [7]. MiRNAs have been demonstrated to be critical regulators of the growth and metastasis of RCC, and are regarded as promising biomarkers and therapeutic targets of RCC [8, 9]. Investigating the expression and function of miRNAs in RCC will contribute to identification of novel biomarkers and therapeutic targets for RCC patients.

MiR-212 is a novel cancer-associated microRNA and has been found to play critical roles in different types of human cancers. It was found to be down-regulated and play tumor suppressive roles in hepatocellular carcinoma [10], ovarian cancer [11], gastric cancer [12] and lung cancer [13, 14]. On the other hand, miR-212 was also found to be overexpressed and play oncogenic roles in pancreatic cancer [15, 16]. However, the expression and function of miR-212 in RCC remain unknown.

In this study, we identified that miR-212 was down-regulated in RCC tissues and cells. MiR-212 inhibited the proliferation, migration and invasion of RCC cells. *In vivo* studies also confirmed that miR-212 inhibited the growth of RCC cells in nude mice. X-linked inhibitor of apoptosis protein (XIAP) was found to be the direct downstream target of miR-212. The expression of XIAP in RCC tissues was negatively correlated with miR-212 level. MiR-212 exerted its tumor suppressive roles in RCC by targeting XIAP. Furthermore, Kaplen-Merier analysis demonstrated that both miR-212 down-regulation and XIAP overexpression predicted the poor prognosis of RCC patients.

## RESULTS

### The expression level of miR-212 is down-regulated in RCC

**Figure 1:**
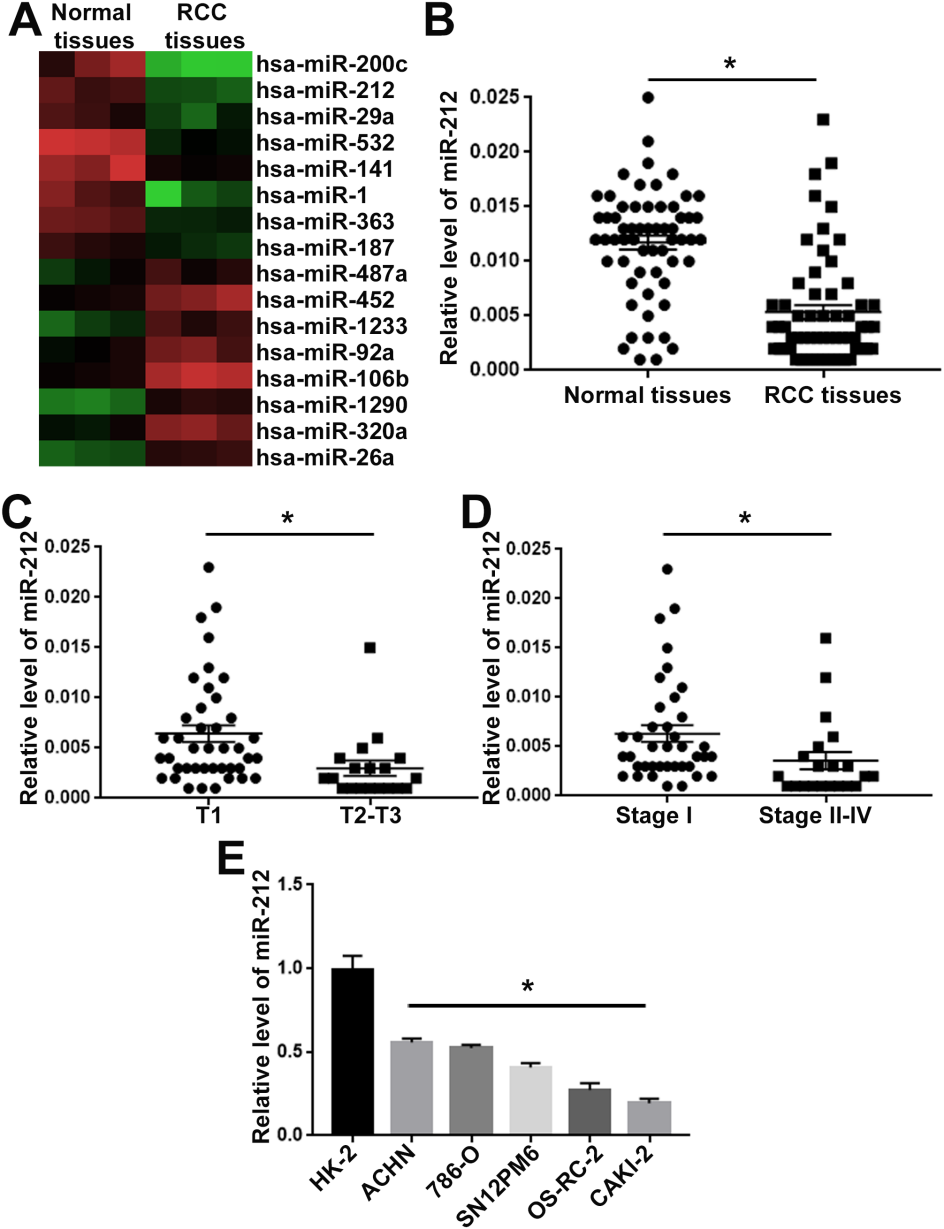
MiR-212 expression was decreased in RCC **(A)** Heatmap for the differentially expressed miRNA in RCC tissues and adjacent non-tumor tissues. MiR-212 was one of the downregulated miRNAs in RCC tissues. **(B)** miR-212 expression was compared between RCC tissues and adjacent non-tumor tissues. The expression of miR-212 was significantly decreased in RCC tissues. **(C)** miR-212 expression was compared between RCC tissues in T1 stage and T2-3 stage. The expression of miR-212 was significantly decreased in RCC tissues of T1 stage. **(D)** miR-212 expression was compared between RCC tissues in TNM I stage and II-IV stage. The expression of miR-212 was significantly decreased in RCC tissues of TNM I stage. **(E)** miR-212 expression was compared between RCC cell lines and HK-2 cells. Compared with HK-2 cells, the expression of miR-212 was significantly decreased in RCC cell lines (ACHN, 786-O, SN12PM6, OS-RC-2 and CAKI-2). ^*^, P<0.05 by t test.

To identify the differentially expressed miRNAs between RCC tissues and adjacent non-tumor tissues, we performed miRNAs screen for these clinical tissues. MiRNA expression signature analysis revealed that 52 miRNAs were downregulated while 39 miRNAs were upregulated (<0.5 fold change) in RCC specimens. The top 8 downregulated (hsa-miR-200c, hsa-miR-212, hsa-miR-29a, hsa-miR-532, hsa-miR-141, hsa-miR-1, hsa-miR-363, hsa-miR-187) and 8 upregulated (hsa-miR-487, hsa-miR-452, hsa-miR-1233, hsa-miR-92a, hsa-miR-106b, hsa-miR-1290, hsa-miR-320, hsa-miR-26a) miRNAs were presented in Figure 1A. Among these miRNAs, miR-212 was one of the top downregualted miRNAs in RCC. To further confirm the miRNA screen results, we performed qRT-PCR for 60 pairs of RCC tissues and adjacent non-tumor tissues. The qRT-PCR confirmed that compared with non-tumor tissues, miR-212 was significantly decreased in RCC tissues (P<0.05, Figure 1B). Furthermore, we confirmed that compared with those in T1 stage, RCC tissues in T2-T3 stages showed significantly decreased level of miR-212 (P<0.05, Figure 1C). Furthermore, the expression level of miR-212 in RCC tissues at stage II-IV was significantly decreased (P<0.05, Figure 1D). Lastly, we evaluated the expression level of miR-212 in RCC cell lines. Compared with HK-2 cells, five RCC cell lines (ACHN, 786-O, SN12PM6, OS-RC-2 and CAKI-2) showed significantly decreased level of miR-212. Taken together, these data indicate miR-212 is significantly decreased in RCC tissues and cells.

### MiR-212 inhibits the proliferation, migration and invasion of RCC cells

**Figure 2:**
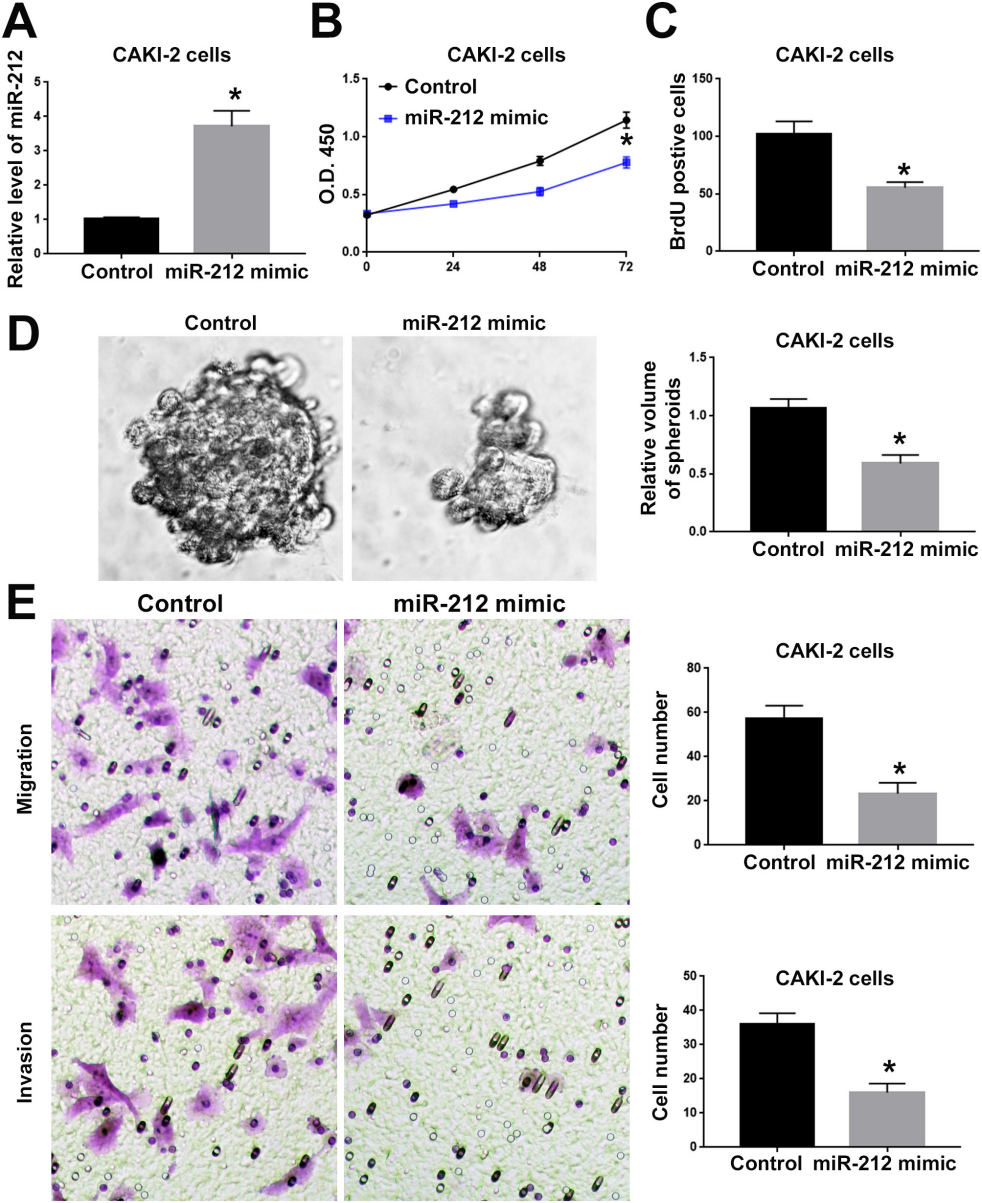
MiR-212 overexpression inhibited the cell viability, proliferation, migration and invasion of CAKI-2 cells **(A)** miR-212 mimic was used to overexpress miR-212 in CAKI-2 cells. Transfection of miR-212 mimic significantly increased the expression of miR-212 in CAKI-2 cells. **(B)** MTS assay was used to evaluate the effect of miR-212 overexpression on cell viability. MiR-212 overexpression significantly inhibited the cell viability of CAKI-2 cells. **(C)** BrdU assay and **(D)** 3D culture was used to investigate the effect of miR-212 overexpression on cell proliferation. MiR-212 overexpression significantly inhibited the cell proliferation of CAKI-2 cells, as suggested by BrdU assay and 3D culture. **(E)** Transwell assay was used to investigate the effect of miR-212 overexpression on cell migration and invasion. MiR-212 overexpression significantly inhibited the cell migration and invasion of CAKI-2 cells. ^*^, P<0.05 by t test and two-way ANOVA.

**Figure 3:**
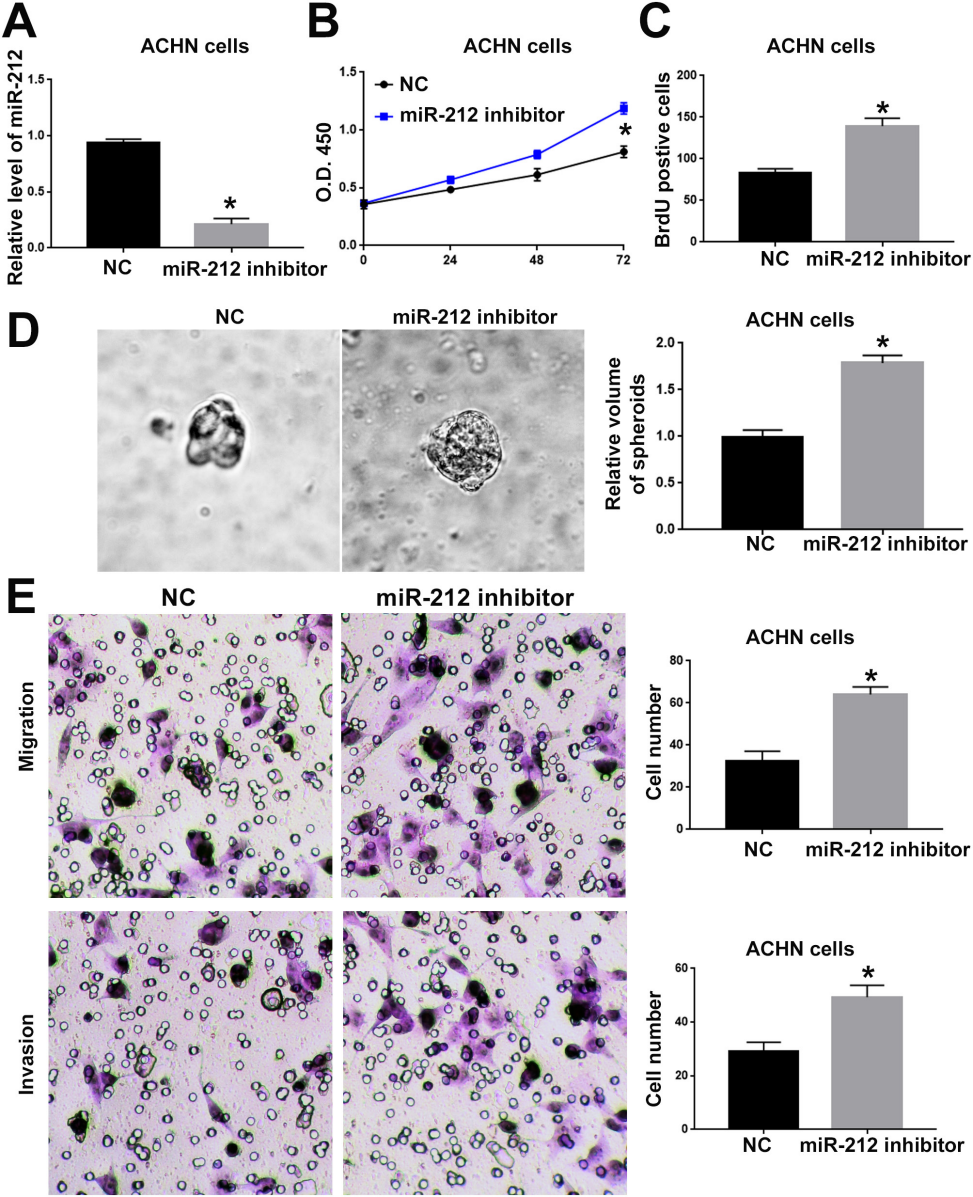
MiR-212 knockdown promoted the cell viability, proliferation, migration and invasion of ACHN cells **(A)** miR-212 inhibitor was used to knockdown miR-212 in ACHN cells. Transfection of miR-212 inhibitor significantly decreased the expression of miR-212 in ACHN cells. **(B)** MTS assay was used to evaluate the effect of miR-212 knockdown on cell viability. MiR-212 knockdown significantly promoted the cell viability of ACHN cells. **(C)** BrdU assay and **(D)** 3D culture was used to investigate the effect of miR-212 knockdown on cell proliferation. MiR-212 knockdown significantly promoted the cell proliferation of ACHN cells, as suggested by BrdU assay and 3D culture. **(E)** Transwell assay was used to investigate the effect of miR-212 knockdown on cell migration and invasion. MiR-212 knockdown significantly promoted the cell migration and invasion of ACHN cells. ^*^, P<0.05 by t test and two-way ANOVA.

**Figure 4:**
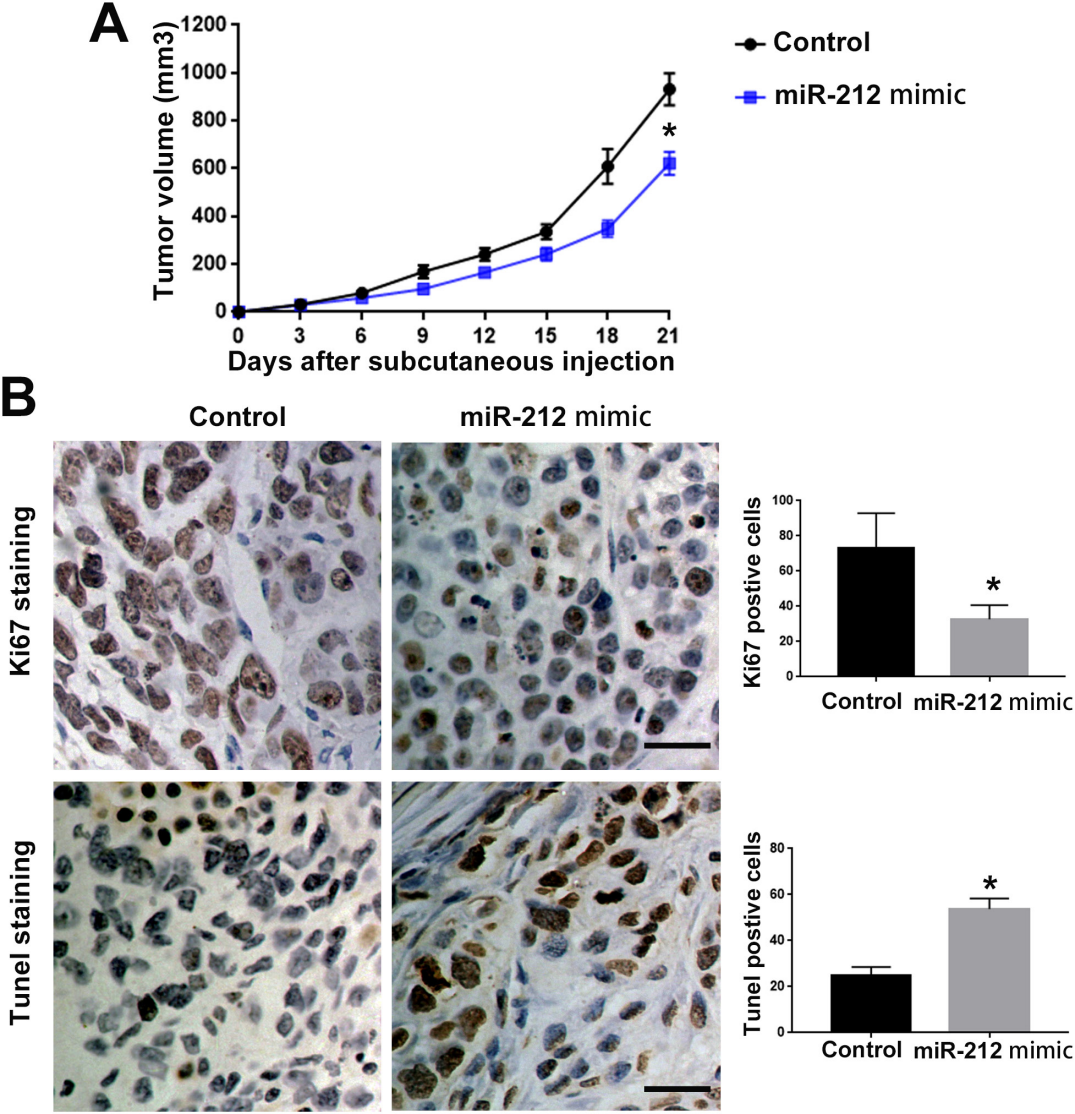
MiR-212 inhibited the *in vivo* growth of RCC cell in nude mice **(A)** CAKI-2 control cells and CAKI cell overexpressing miR-212 were subcutaneous injected into nude mice to investigate the effect of miR-212 on *in vivo* growth of RCC cells. miR-212 overexpression inhibited the *in vivo* growth of CAKI-2 cells in nude mice. **(B)** miR-212 overexpression reduced the Ki-67 postive cells and increased Tunel postive cells in subcutaneous tumors. Scale bar: 40um; ^*^, P<0.05 by t test and two-way ANOVA.

To investigate the functional role of miR-212 in RCC, we overexpressed miR-212 in CAKI-2 cells. Transfection of miR-212 overexpressing vector into CAKI-2 cells significantly increased miR-212 level in CAKI-2 cells (P<0.05, Figure 2A). Overexpression of miR-212 significantly decreased the cell viability of CAKI-2 cells (P<0.05, Figure 2B). BrdU and 3D culture demonstrated that compared with control vector, miR-212 overexpressing vector significantly decreased the proliferative ability of CAKI-2 cells (P<0.05, Figure 2C and 2D). Furthermore, Transwell assay confirmed that forced expression of miR-212 significantly reduced the migration (P<0.05, Figure 2E) and invasion (P<0.05, Figure 2F) of CAKI-2 cells. On the other hand, miR-212 inhibitor significantly decreased the expression of miR-212 in ACHN cells (P<0.05, Figure 3A). Knockdown of miR-212 resulted in increased cell viability (P<0.05, Figure 3B), proliferation (P<0.05, Figure 3C and 3D), migration (P<0.05, Figure 3E) and invasion (P<0.05, Figure 3F) of ACHN cells. Addtionally, we examined the effect of miR-212 on the Caspase3/7 activity on RCC cells. The data in Supplementary Figure 1A and 1B showed that miR-212 overexpression increased the Caspase3/7 activity while its knockdown decreased the Caspase3/7 activity (P<0.05). To further investigate the oncogenic effects of miR-212 on RCC cells, we performed subcutaneous injection of RCC cells in nude mice. Compared with CAKI-2 cells transfected with control vector, CAKI-2 cells overexpressing miR-212 showed significantly decreased *in vivo* growth ability (P<0.05, Figure 4A). Ki67 staining and Tunel staining confirmed that miR-212 overexpression significantly decreased the proliferation (P<0.05, Figure 4B) and decreased the apoptosis (P<0.05, Figure 4B) of CAKI-2 cells in nude mice. Therefore, these data indicate that miR-212 plays tumor-suppressive role in RCC by inhibiting the proliferation, migration and invasion of RCC cells.

### XIAP is the downstream target of miR-212 in RCC

**Figure 5:**
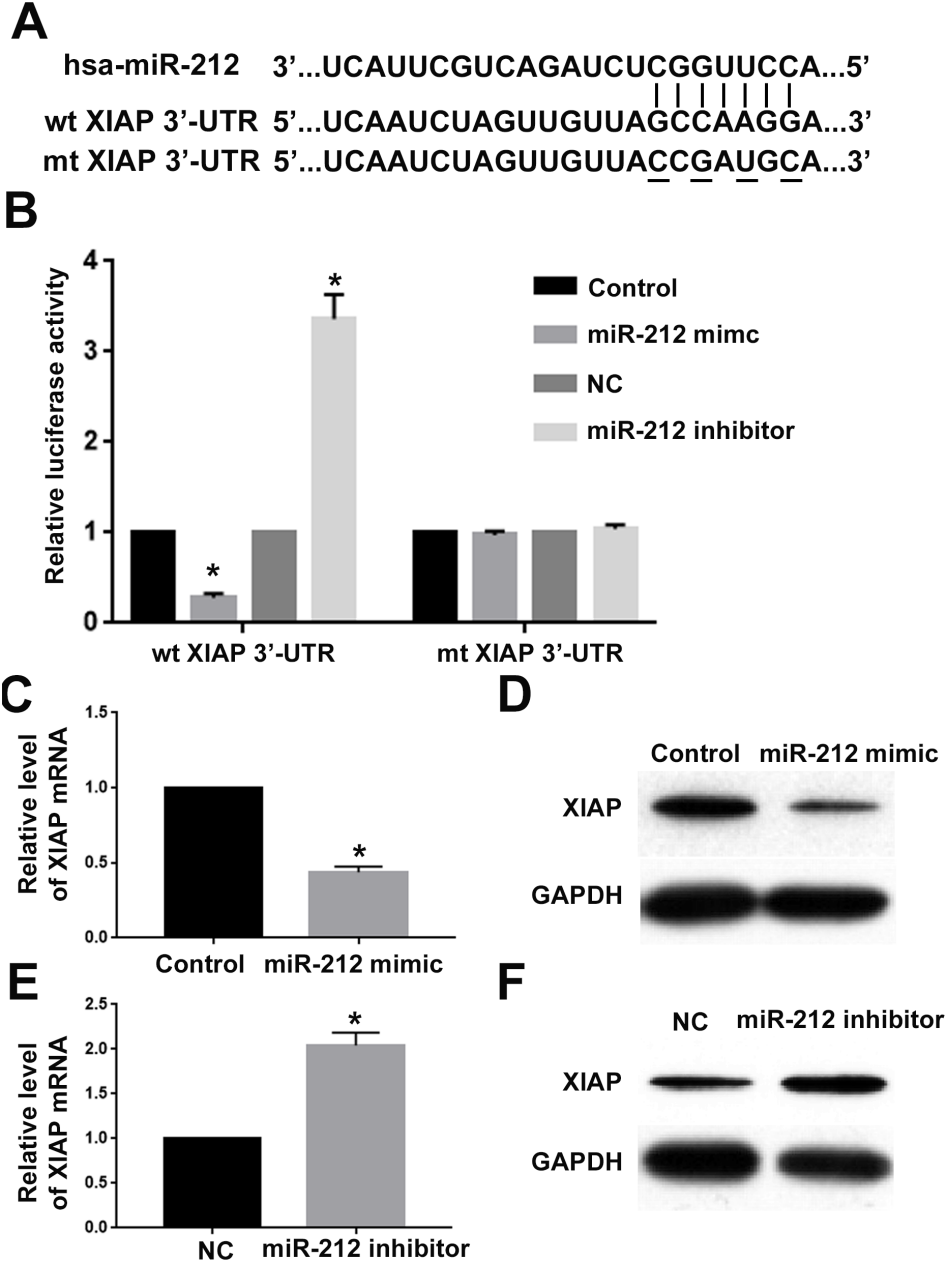
XIAP was the downstream target of miR-212 in RCC **(A)** MiR-212 and its putative binding sequence in the 3′-UTR of XIAP. Mutated XIAP 3′-UTR was generated in the complementary site for the seed region of miR-212. **(B)** miR-212 overexpression significantly suppressed the luciferase activity of wild type XIAP 3′-UTR but not mutated 3′-UTR of XIAP. Downregulating miR-212 increased the luciferase activity of wild type XIAP 3′-UTR but not mutated 3′-UTR of XIAP. **(C)** MiR-212 overexpression significantly inhibited the mRNA level of XIAP in CAKI-2 cells. **(D)** MiR-212 overexpression significantly inhibited the protein level of XIAP in CAKI-2 cells. **(E)** MiR-212 inhibition significantly increased the mRNA level of XIAP in ACHN cells. **(F)** miR-212 inhibition significantly increased the protein level of XIAP in ACHN cells. ^*^, P<0.05 by t test.

**Figure 6:**
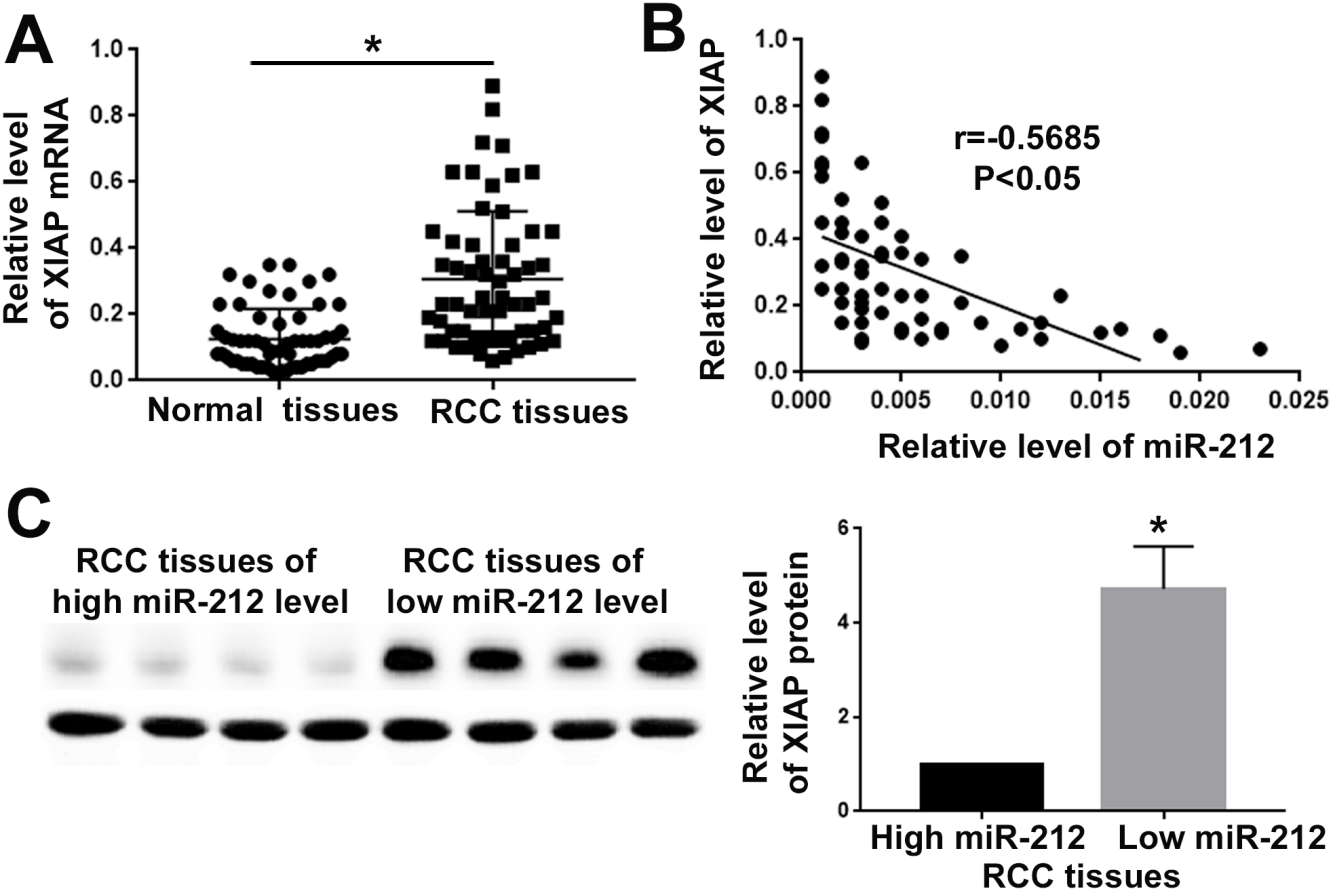
MiR-212 level was negatively correlated with XIAP expression in RCC tissues **(A)** XIAP expression was compared between RCC tissues and adjacent non-tumor tissues. The expression of XIAP was significantly increased in RCC tissues. **(B)** The correlation analysis was performed for miR-212 mRNA and XIAP mRNA in RCC tissues. MiR-212 level was negatively correlated with XIAP expression level in RCC tissues. **(C)** Western blot was used to compare the XIAP level between RCC tissues with low and high level of miR-212. XIAP level was significantly decreased in RCC tissues with high level of miR-212 ^*^, P<0.05 by t test and Spearman's correlation analysis.

To clarify the molecular mechanisms underlying the tumor suppressive roles of miR-212 in RCC, we used the public database to search for the potentially downstream targets of miR-212. X-linked inhibitor of apoptosis protein (XIAP), a critical regulator of cell proliferation, apoptosis and motility [17–19], was predicted to be a potentially downstream target of miR-212. As shown in Figure 5A, XIAP 3′-UTR contained the complementary sequences for miR-212 binding. To validate miR-212 could indeed bind with the 3′-UTR of XIAP, we performed luciferase activity assay for wild type XIAP 3′-UTR and mutated XIAP 3′-UTR. Overexpression of miR-212 significantly decreased while miR-212 knockdown significantly increased the luciferase activity of wild type XIAP 3′-UTR (P<0.05, Figure 6B). In contrast, miR-212 overexpression or knockdown did not have any effect on the luciferase activity of mutated XIAP 3′-UTR (Figure 6B). Furthermore, qRT-PCR and western blot confirmed that miR-212 overexpression decreased the mRNA (P<0.05, Figure 5C) and protein (P<0.05, Figure 5D) level of XIAP in CAKI-2 cells. MiR-212 knockdown increased the mRNA (P<0.05, Figure 5E) and protein (P<0.05, Figure 5F) level of XIAP in CAKI-2 cells. These data demonstrate that miR-212 could inhibit the expression of XIAP in RCC cells by interacting with the 3′-UTR of XIAP.

### IAP expression is negatively correlated with miR-212 level in RCC tissues

To further confirm the regulatory effect of miR-212 on XIAP, we measured the expression level of XIAP in RCC tissues. qRT-PCR demonstrated that XIAP mRNA level was significantly increased in RCC tissues (P<0.05, Figure 6A). Correlation analysis for XIAP and miR-212 showed that the level of miR-212 was negatively correlated with the XIAP level in RCC tissues (P<0.05, Figure 6B). Western blot further demonstrated that in RCC tissues with high miR-212 level, the expression of XIAP was significantly decreased, compared with the tissues of low miR-212 level (P<0.05, Figure 6C). Taken together, these data demonstrate that miR-212 can inhibit XIAP expression in RCC.

### XIAP mediates the tumor suppressive functions of miR-212 in RCC

**Figure 7:**
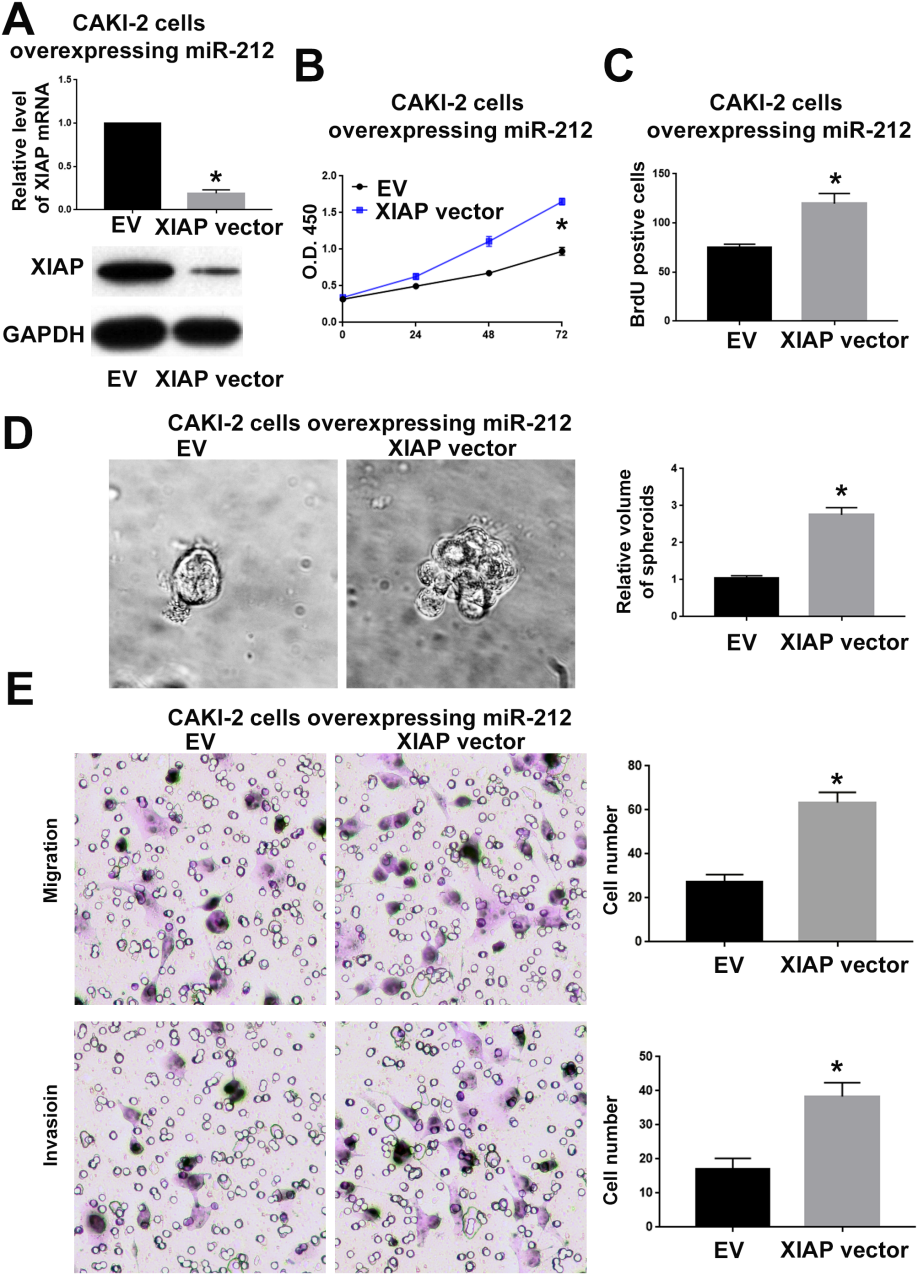
XIAP overexpression reversed the inhibitory effect of miR-212 overexpression on CAKI-2 cells **(A)** The XIAP vector significantly increased the mRNA and protein level of XIAP in CAKI-2 overexpressing miR-212. **(B**-**E)** XIAP overexpression reversed the inhibitory effect of miR-212 overexpression on the viability (B), proliferation (C & D), migration and invasion (E) of CKAI-2 cells. ^*^P < 0.05 by t test and two-way ANOVA.

**Figure 8:**
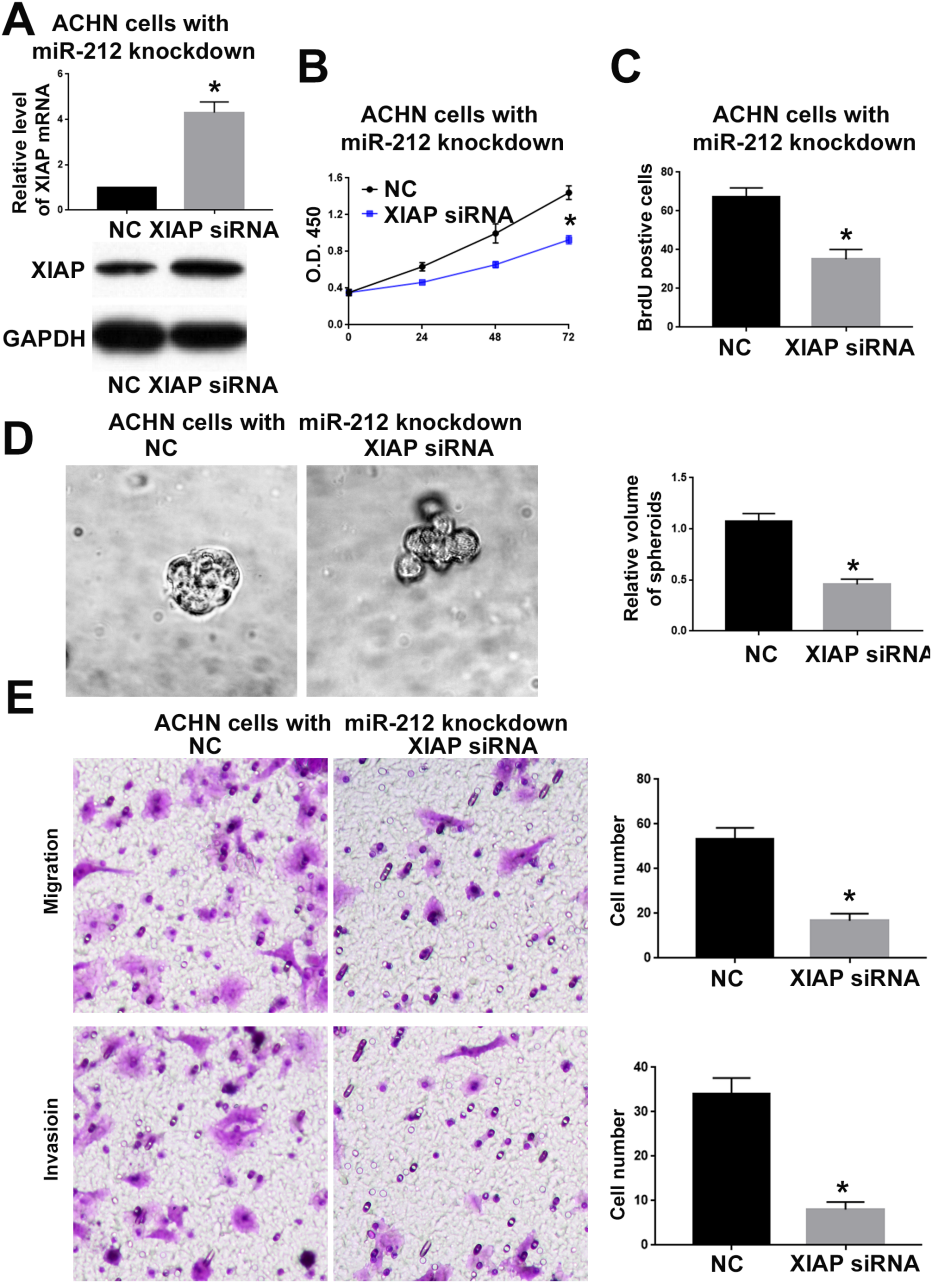
XIAP knockdown abrogated the promoting effect of miR-212 knockdown on ACHN cells **(A)** The XIAP shRNA significantly decreased the mRNA and protein level of XIAP in ACHN cells with miR-212 knockdown. **(B**-**E)** XIAP knockdown abrogated the promoting effect of miR-212 knockdown on the viability (B), proliferation (C & D), migration and invasion (E) of CKAI-2 cells. ^*^P < 0.05 by t test and two-way ANOVA.

To confirm whether XIAP is critical for the function of miR-212 in RCC, we overexpressed XIAP in CAKI-2 cells overexpressing miR-212 and investigated whether restoring XIAP expression could reverse the inhibitory effect of miR-212 on CAKI-2 cells. As shown in Figure 7A, XIAP vector significantly increased XIAP expression in in CAKI-2 cells overexpressing miR-212 (P<0.05). Functionally, overexpressing XIAP reversed the inhibitory effects of miR-212 on cell viability (P<0.05, Figure 7B), proliferation (P<0.05, Figure 7C and 7D), migration (P<0.05, Figure 7F) and invasion (P<0.05, Figure 7F). Moreover, inhibiting XIAP expression in ACHN cells (P<0.05, Figure 8A) abrogated the promoting effect of miR-212 knockdown on cell viability (P<0.05, Figure 8B), proliferation (P<0.05, Figure 8C), migration (P<0.05, Figure 8D) and invasion (P<0.05, Figure 8E). These data indicate that XIAP is not only a downstream target of miR-212, but also a functional mediator of miR-212 in RCC.

### MiR-212 downregulation and XIAP overexpression predict poor prognosis of RCC

**Figure 9:**
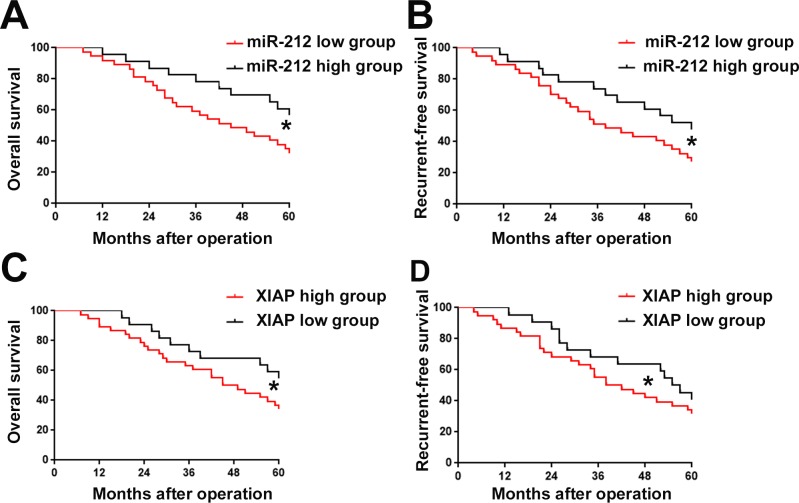
The prognostic value of miR-212 and XIAP for RCC patients assessed Kaplen-Merier analysis was performed to investigate the prognostic value of miR-212 and XIAP for RCC patients. RCC patients with low expression level of miR-212 had significantly decreased rate of Overall survival (OS) **(A)** and Recurrent-free survival (RFS) **(B)**. RCC patients with high expression level of miR-212 had significantly decreased rate of OS **(C)** and RFS **(D)**.

After confirming the expression and function of miR-212 and XIAP in RCC, we lastly examined whether aberrant expression of miR-212 and its downstream target XIAP could predict the prognosis of RCC patients. The mean expression level of miR-212 and XIAP was defined as cutoff value to divide RCC patients into: miR-212 low group and high group, and, XIAP low group and high group. As shown in Figure 9A and 9B, decreased expression of miR-212 was correlated with decreased rate of overall survival (OS) (P<0.05) and recurrent-free survival (RFS) (P<0.05). Furthermore, Kaplen-Merier analysis also demonstrated that patients with increased expression of XIAP had significantly decreased rate of OS (P<0.05, Figure 9C) and RFS (P<0.05, Figure 9D). These indicate that miR-212 and XIAP can both serve as prognosis predictor for RCC patients.

## DISCUSSION

Accumulating studies have demonstrated miRNAs actively participated in the development and progression of various human cancers [20]. Identifying cancer-associated miRNAs and their biological functions in cancer cells can promote the identification of biomarkers and therapeutic targets for human cancers. In this study, we used miRNAs screen assay to identify the differentially expressed miRNAs between RCC tissues and non-tumor tissues. Among numerous miRNAs, miR-212 was one of the significantly downregualted miRNAs in RCC tissues. qRT-PCR further confirmed that the expression of miR-212 was significantly decreased in RCC tissues. In RCC tissues of advanced T stage and TNM stage, the level of miR-212 was significantly decreased. Furthermore, we demonstrated that miR-212 was significantly decreased in RCC cell lines. These data demonstrate that miR-212 is a tumor suppressive microRNA in RCC. Functionally, miR-212 was found to inhibit the growth of hepatocellular carcinoma by targeting FOXA1 [10]. Study of gastric cancer also demonstrated that miR-212 inhibited the proliferation of gastric cancer cells by inhibiting [21]. However, study of pancreatic cancer cells found that miR-212 promoted cell proliferation by targeting patched-1 [16]. In this study we demonstrated that overexpression of miR-212 could inhibit the viability, proliferation, migration and invasion of RCC cells while miR-212 knockdown promoted these biological functions of RCC cells. Therefore, miR-212 exerted its tumor suppressive roles in RCC by inhibiting cell viability, proliferation, migration and invasion.

To clarify the detailed mechanisms by which miR-212 exerts tumor suppressive effects in RCC cells, we further investigated the downstream target of miR-212. XIAP, an important regulator of cell apoptosis pathway, has been found to be aberrantly expressed in various types of human cancers [22–25]. Poorly differentiated carcinomas showed significantly elevated levels of XIAP than well differentiated carcinomas. XIAP expression in metastatic tissues was also significantly higher than that in primary tumor sites [26]. XIAP could inhibit the function of caspase-3, 7 and 9, and thus prevent cell apoptosis [27–29]. Interestingly, XIAP was also found to interact with the Rho GDP dissociation inhibitor (RhoGDI) to promtoe the cell motility [19]. In this study, qRT-PCR, western blot and luciferase activity assay demonstrated that XIAP was the direct downstream target of miR-212 in RCC. More importantly, XIAP was found to mediate the functional influence of miR-212 on cell viability, proliferation, migration and invasion. Therefore, these data indicate that miR-212 inhibits the proliferation, migration and invasion of RCC cells by targeting XIAP protein, and can potentially serve as a promising therapeutic target for RCC. It is worth to mention here that one miRNA potentially has multiple downstream targets. Previous stduies showed FOXA1, HBEGF, MECP2 and PED were the downstream targets of miR-212 in different types of cells. It is worth to investigate in the future whether the regulatory effects of miR-212 on these protein can be observed in RCC cells.

Increasing studies showed that miRNAs could serve as promising prognostic indicators of human cancers [30]. Therefore, we evaluated the prognostic value of miR-212 in RCC. Decreased expression level of miR-212 was correlated with poor prognosis of RCC patients. Additionally, XIAP overexpression in RCC was correlated with poor prognosis of RCC patients. These indicate that miR-212 and XIAP are promising prognostic predictors for RCC patients. It will be of great importance in the future to investigate whether miR-212 and XIAP can be employed as used as biomarkers for RCC early diagnosis.

In summary, this study finds that miR-212 is down-regulated in RCC and its decreased expression is associated with RCC progression. *In vitro* and *in vivo* studies confirm that miR-212 inhibit the proliferation, migration and invasion of RCC cells. Furthermore, we identify that XIAP protein was the direct downstream target of miR-212. Lastly, miR-212 and XIAP are prognostic predictors for RCC patients.

## MATERIALS AND METHODS

### Clinical tissues

60 RCC samples and adjacent non-tumor tissues were collected after getting informed consent from the RCC patients. All patients received partial or radical nephrectomy in the Department of Departments of Urology and Henan Institute of Urology during January 2005 to December 2010. The clinical tissues were stored at liquid nitrogen after surgical resection. All protocols involving clinical tissues were approved by the Ethics Committee of Zhengzhou University.

### Screen for differentially expressed miRNAs between RCC tissues and non-tumor tissues

TaqMan LDA Human microRNA Panel v2.0 (Applied Biosystems, Foster City, CA, USA) was used to identify the differentiated miRNAs between RCC tissues and adjacent non-tumor tissues. Three pairs of RCC tissues and adjacent non-tumor tissues were used for miRNAs screen. After generating cDNAs by reverse transcription, the TaqMan real-time PCR assay for these cDNAs was performed. GeneSpring GX software (version 7.3.1, Agilent Technologies) was used to analyze the relative miRNA expression. P < 0.05 was used to define the differentially expressed miRNAs.

### Cell culture and transfection

Five RCC cell lines (ACHN, 786-O, SN12PM6, OS-RC-2 and CAKI-2) and human proximal tubule epithelial cell line HK-2 cell line were obtained from American Type Culture Collection (ATCC, Manassas, VA, USA). RCC cells were cultured in RPMI 1640 medium (Gibco, Grand Island, NY, USA) supplemented with 10% fetal bovine serum (FBS, Gibco), 100 units/mL penicillin and 100 μg/mL streptomycin (Sigma, St-Louis, MO, USA). HK-2 cells were maintained in complete RPMI 1640 medium supplemented with keratinocyte serum-free medium, bovine pituitary extract, and human recombinant epidermal growth factor (Invitrogen, Carlsbad, CA, USA).

MiR-212 overexpressing vector, the control vector, miR-212 inhibitor and the negative control were bought from the company of Genecopoeia (Guangzhou, China). Plasmids expressing XIAP were purchased from Addgene (Cambridge, MA, USA). XIAP specific shRNAs were purchased from OriGene (Beijing, China). These vectors were transfected into RCC cells using Lipofectamine 2000 according to the manufacturer's instructions (Invitrogen, Carlsbad, CA, USA).

### MTS assay and BrdU assay

Cell viability was determined by MTS assay (Roche, USA) performed according to the manufacturer's instructions. The proliferative ability of RCC cells was determined by BrdU (5-bromodeoxyuridine) (chemiluminescent) (Roche, USA) assay based on manufacturer's instructions.

### 3D culture for cancer cells

300 μl of Matrigel was added to each well of 24-well plates. After the gel was solidified, 5000 RCC cells suspended in 1mL RPMI 1640 medium were seeded into each well and 3D spheroids were formed after culturing the cells for 5 days. The spheroids were observed under the inverted microscope and pictures were captured. Relative sizes of these spheroids were compared using Image J software.

### Migration and invasion assays

Cell migration and invasion were evaluated by Transwell assay. RCC cells were starved in basal medium for 24 hours before transwell assay. For invasion assay, matrigel diluted in basal medium (1:8 dilution) was used to coat the membrane of upper chamber. RCC cells suspended in basal medium were seeded into the upper chamber. 10% FBS used as chemoattractant was added into the bottom chamber. 12-24 hours later, RCC cells migrated or invaded through the chamber membrane were fixed with methanol and stained with crystal violet solution.

### qRT-PCR

Total RNA was extracted from RCC tissues and cultured RCC cells using miRNeasy Mini Kit or RNeasy Mini Kit (Qiagen). Reverse Transcription for cDNA was performed using TaqMan MicroRNA Reverse Transcription Kit (Applied Biosystems) or iScript^™^ cDNA Synthesis Kit (BIORAD). TaqMan Human MiRNA Assay Kit (Applied Biosystems) was used to evaluate the relative expression of miR-212 [31]. Platinum SYBR Green qPCR Supermix UDG kit (Invitrogen, Carlsbad, CA) was used to evaluate the relative level of XIAP. GAPDH and U6 were used as the internal control for XIAP and miR-212, respectively.

### Western blotting analysis

Cellular protein were extracted from RCC tissues and cells using RIPA buffer (Thermo Scientifc, Rockford, IL, USA) supplemented with protease inhibitor (Beyotime Institute of Biotechnology, Haimen, China) and PMSF (Beyotime Institute of Biotechnology). Thirty micrograms of protein lysate were separated in 4-20% SDS-PAGE gels and transferred onto PVDF membranes (Beyotime Institute of Biotechnology). The membranes were incubated with primary antibodies including XIAP and GAPDH at 4°C overnight. Secondary antibodies conjugated with horseradish peroxidase (Boster Bio-engineering Limited Company, Wuhan, China) were incubated with the membranes for 2 hrs at room temperature. The protein signals were dectected using ECL kit (Beyotime Institute of Biotechnology).

### Luciferase reporter assay

Wild type (wt) XIAP 3′-UTR sequence or the mutated (mt) XIAP 3′-UTR sequences were inserted into the pGL3 vector (Promega, Madison, WI, USA). ACHN cells in 24-well plates were cultured in OptimMEM reduced serum media (Life Technologies), and were transfected with luciferase reporter construct (the wt or mt 3′-UTR of XIAP) and miR-212 overexpressing vector, miR-212 inhibitor, corresponding control vectors or negative control vectors using Fugene (Promega, Madison, WI, USA). 24 hrs later, luciferase activity was evaluated by the luciferase reporter assay system (Promega, Madison, WI, USA).

### *In vivo* experiments

BALB/c nude mice were used to establish the nude mouse xenograft model. CAKI-2 cells transfected with miR-212 overexpressing vector or control vectors were suspended in 100 uL PBS and were injected subcutaneously into the flank of nude mouse. The width and length of the tumor nodules were measured every three days. The volume of subcutaneous tumors was calculated as tumor volume = length × width × width/2. 3 weeks later, the xenograft tumor tissues were explanted for Ki67 staining (Cell Signaling, Danvers, MA, USA) and Tunel staining assay. *In vivo* protocols were approved by the Institutional Animal Care and Use Committee of Zhengzhou University.

### Statistical analysis

The data in this study are presented as mean ± S.E.M. Statistical analysis including Pearson chi-squared test, a two-tailed Student's t test, a Kaplen-Merier plot and the ANOVA test were performed using SPSS software (Version 13, SPSS, Chicago, IL, USA) and GraphPad Prism software (GraphPad Software, Inc, San Diego, CA, USA). P < 0.05 was regarded to be statistically significant

## SUPPLEMENTARY MATERIALS FIGURE



## Abbreviations

RCC: renal cell carcinoma
qRT-PCR: quantitative real time polymerase chain reaction

## References

[1] Cohen HT, McGovern FJ (2005). Renal-cell carcinoma. N Engl J Med.

[2] Cheville JC, Lohse CM, Zincke H, Weaver AL, Blute ML (2003). Comparisons of outcome and prognostic features among histologic subtypes of renal cell carcinoma. Am J Surg Pathol.

[3] Ljungberg B, Cowan NC, Hanbury DC, Hora M, Kuczyk MA, Merseburger AS, Patard JJ, Mulders PF, Sinescu IC (2010). EAU guidelines on renal cell carcinoma: the 2010 update. European urology.

[4] Skinner DG, Colvin RB, Vermillion CD, Pfister RC, Leadbetter WF (1971). Diagnosis and management of renal cell carcinoma: a clinical and pathologic study of 309 cases. Cancer.

[5] Bartel DP (2004). MicroRNAs: genomics, biogenesis, mechanism, and function. Cell.

[6] Bartel DP (2009). MicroRNAs: target recognition and regulatory functions. Cell.

[7] Zimmerman AL, Wu S (2011). MicroRNAs, cancer and cancer stem cells. Cancer Lett.

[8] Tsz-Fung FC, Youssef YM, Lianidou E, Romaschin AD, Honey RJ, Stewart R, Pace KT, Yousef GM (2010). Differential expression profiling of microRNAs and their potential involvement in renal cell carcinoma pathogenesis. Clin Biochem.

[9] Weng L, Wu X, Gao H, Mu B, Li X, Wang JH, Guo C, Jin JM, Chen Z, Covarrubias M (2010). MicroRNA profiling of clear cell renal cell carcinoma by whole - genome small RNA deep sequencing of paired frozen and formalin - fixed, paraffin - embedded tissue specimens. J Pathol.

[10] Dou C, Wang Y, Li C, Liu Z, Jia Y, Li Q, Yang W, Yao Y, Liu Q, Tu K (2015). MicroRNA-212 suppresses tumor growth of human hepatocellular carcinoma by targeting FOXA1. Oncotarget.

[11] Wei LQ, Liang HT, Qin DC, Jin HF, Zhao Y, She MC (2014). MiR-212 exerts suppressive effect on SKOV3 ovarian cancer cells through targeting HBEGF. Tumor Biol.

[12] Wada R, Akiyama Y, Hashimoto Y, Fukamachi H, Yuasa Y (2010). miR-212 is downregulated and suppresses methyl - CpG - binding protein MeCP2 in human gastric cancer. Int J Cancer.

[13] Incoronato M, Urso L, Portela A, Laukkanen MO, Soini Y, Quintavalle C, Keller S, Esteller M, Condorelli G (2011). Epigenetic regulation of miR-212 expression in lung cancer. PLoS One.

[14] Incoronato M, Garofalo M, Urso L, Romano G, Quintavalle C, Zanca C, Iaboni M, Nuovo G, Croce CM, Condorelli G (2010). miR-212 increases tumor necrosis factor–related apoptosis-inducing ligand sensitivity in non–small cell lung cancer by targeting the antiapoptotic protein PED. Cancer Res.

[15] Park JK, Henry JC, Jiang J, Esau C, Gusev Y, Lerner MR, Postier RG, Brackett DJ, Schmittgen TD (2011). miR-132 and miR-212 are increased in pancreatic cancer and target the retinoblastoma tumor suppressor. Biochem Biophys Res Commun.

[16] Ma C, Nong K, Wu B, Dong B, Bai Y, Zhu H, Wang W, Huang X, Yuan Z, Ai K (2014). miR-212 promotes pancreatic cancer cell growth and invasion by targeting the hedgehog signaling pathway receptor patched-1. J Exp Clin Cancer Res.

[17] Riedl SJ, Renatus M, Schwarzenbacher R, Zhou Q, Sun C, Fesik SW, Liddington RC, Salvesen GS (2001). Structural basis for the inhibition of caspase-3 by XIAP. Cell.

[18] Cummins JM, Kohli M, Rago C, Kinzler KW, Vogelstein B, Bunz F (2004). X-linked inhibitor of apoptosis protein (XIAP) is a nonredundant modulator of tumor necrosis factor-related apoptosis-inducing ligand (TRAIL)-mediated apoptosis in human cancer cells. Cancer Res.

[19] Liu J, Zhang D, Luo W, Yu Y, Yu J, Li J, Zhang X, Zhang B, Chen J, Wu XR (2011). X-linked inhibitor of apoptosis protein (XIAP) mediates cancer cell motility via Rho GDP dissociation inhibitor (RhoGDI)-dependent regulation of the cytoskeleton. J Biol Chem.

[20] Volinia S, Calin GA, Liu CG, Ambs S, Cimmino A, Petrocca F, Visone R, Iorio M, Roldo C, Ferracin M (2006). A microRNA expression signature of human solid tumors defines cancer gene targets. Proc Natl Acad Sci U S A.

[21] Jiping Z, Ming F, Lixiang W, Xiuming L, Yuqun S, Han Y, Zhifang L, Yundong S, Shili L, Chunyan C (2013). MicroRNA - 212 inhibits proliferation of gastric cancer by directly repressing retinoblastoma binding protein 2. J Cell Biochem.

[22] Cao C, Mu Y, Hallahan DE, Lu B (2004). XIAP and survivin as therapeutic targets for radiation sensitization in preclinical models of lung cancer. Oncogene.

[23] Nemoto T, Kitagawa M, Hasegawa M, Ikeda S, Akashi T, Takizawa T, Hirokawa K, Koike M (2004). Expression of IAP family proteins in esophageal cancer. Exp Mol Pathol.

[24] Nagi C, Xiao GQ, Li G, Genden E, Burstein DE (2007). Immunohistochemical detection of X-linked inhibitor of apoptosis in head and neck squamous cell carcinoma. Ann Diagn Pathol.

[25] Mizutani Y, Nakanishi H, Li YN, Matsubara H, Yamamoto K, Sato N, Shiraishi T, Nakamura T, Mikami K, Okihara K (2007). Overexpression of XIAP expression in renal cell carcinoma predicts a worse prognosis. Int J Oncol.

[26] Gordon G, Mani M, Mukhopadhyay L, Dong L, Edenfield H, Glickman J, Yeap B, Sugarbaker D, Bueno R (2007). Expression patterns of inhibitor of apoptosis proteins in malignant pleural mesothelioma. J Pathol.

[27] Asselin E, Mills GB, Tsang BK (2001). XIAP regulates Akt activity and caspase-3-dependent cleavage during cisplatin-induced apoptosis in human ovarian epithelial cancer cells. Cancer Res.

[28] Chai J, Shiozaki E, Srinivasula SM, Wu Q, Datta P, Alnemri ES, Shi Y (2001). Structural basis of caspase-7 inhibition by XIAP. Cell.

[29] Shiozaki EN, Chai J, Rigotti DJ, Riedl SJ, Li P, Srinivasula SM, Alnemri ES, Fairman R, Shi Y (2003). Mechanism of XIAP-mediated inhibition of caspase-9. Mol Cell.

[30] Calin GA, Croce CM (2006). MicroRNA-cancer connection: the beginning of a new tale. Cancer Res.

[31] Gu CH, Tian FY, Pu JR, Zheng LD, Mei H, Zeng FQ, Yang JJ, Kan QC, Tong QS (2014). Over-expression of testis-specific expressed gene 1 attenuates the proliferation and induces apoptosis of GC-1spg cells. J Huazhong Univ Sci Technolog Med Sci.

