# Supplementary material for: MicroRNA-212 inhibits the proliferation, migration and invasion of renal cell carcinoma by targeting X-linked inhibitor of apoptosis protein (XIAP)
Source: Oncotarget. 2017 Sep 8;8(54):92119–33. doi: 10.18632/oncotarget.20786 (PMC5696168; doi:10.18632/oncotarget.20786)
Supplement: Supplementary file 1 [file oncotarget-08-92119-s001.pdf]

MicroRNA-212 inhibits the proliferation, migration and invasion of renal cell carcinoma by targeting X-linked inhibitor of apoptosis protein (XIAP)

SUPPLEMENTARY MATERIALS

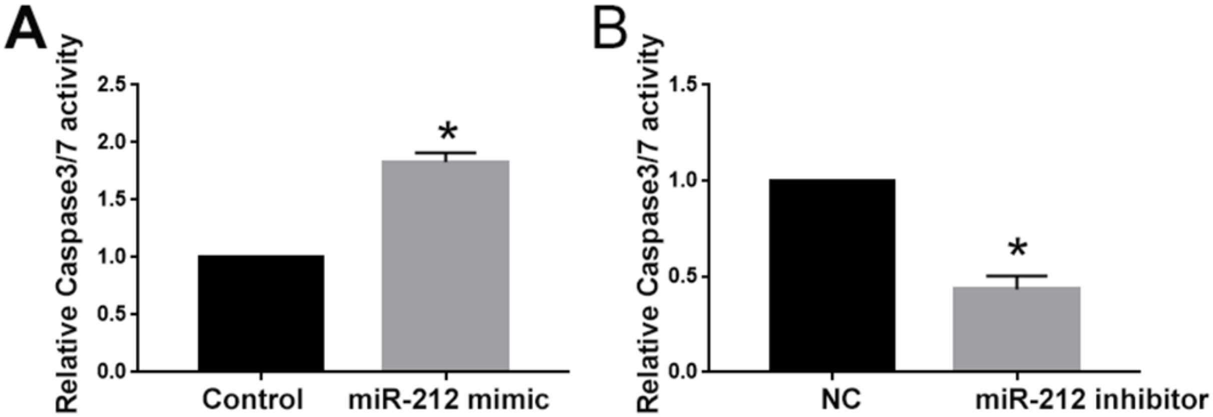

Supplementary Figure 1: The effect of miR-212 on Caspase3/7 activity of RCC cells. **(A)** Overexpression of miR-212 increased the Caspase3/7 activity in CAKI-2 cells. **(B)** Knockdown of miR-212 decreased the Caspase3/7 activity in ACHN cells. $^{*}P < 0.05$ by t test.
